# Concussion Risk and the Need for Prevention: An Exploration into the Complexity of Community Perspectives in Rugby Union

**DOI:** 10.1007/s40279-025-02243-0

**Published:** 2025-06-19

**Authors:** Marelise Badenhorst, Janelle Romanchuk, Danielle Salmon, James Craig Brown, Sharief Hendricks, Simon Walters

**Affiliations:** 1 School of Sport and Recreation, Sports Performance Research Institute New Zealand, Auckland University of Technology, Auckland, New Zealand; 2 Carnegie Applied Rugby Research (CARR) Centre, Carnegie School of Sport, Leeds Beckett University, Leeds, UK; 3 New Zealand Rugby, Wellington, New Zealand; 4 Division of Physiological Sciences and Health Through Physical Activity, Lifestyle and Sport Research Centre, Department of Human Biology, Faculty of Health Sciences, University of Cape Town, Cape Town, South Africa; 5 Institute of Sport and Exercise Medicine, Department of Exercise, Sport and Lifestyle Medicine, Faculty of Medicine and Health Sciences, Stellenbosch University, Stellenbosch, South Africa

## Abstract

**Background:**

Community perceptions of injury risk can impact participation rates and may influence attitudes and behaviours around prevention efforts. Understanding how end-users think about concussion-related risk and the need for prevention is critical for the design and implementation of interventions. This study aimed to explore community rugby union stakeholders’ perceptions of concussion risk and the need for prevention.

**Methods:**

This pragmatic, qualitative descriptive study utilised semi-structured interviews and focus groups with 62 school- and club-level community rugby stakeholders (provincial union representatives, players, coaches, school/club sport administrators, team leads (managers), physiotherapists, nurses and doctors) from across New Zealand during the 2022 rugby season. Reflexive thematic analysis was used to analyse the data.

**Results:**

Three themes were developed from the data. The theme ‘concussion risk: a spectrum of concern’ included a spectrum of beliefs ranging from ‘concussions are a problem’ to the belief that concussions are only a problem if not managed well or that it has always been part of the game, and the risks are being exaggerated. A second theme, ‘focus on technique and conditioning, or is injury just the nature of the game?’, described beliefs in the importance of technique and conditioning or contrary beliefs such as ‘as long as there is contact, there will be concussion’. A third theme, ‘conflicting concussion narratives’, described the tendency of some participants to move back and forth across the spectrum of risk and prevention perceptions, depending on the context.

**Conclusions:**

Findings reveal a diversity of perspectives on how concussions should be managed or prevented. Balancing these perspectives is critical. This involves addressing unfavourable beliefs, prioritizing both prevention and effective management and community-wide education.

**Supplementary Information:**

The online version contains supplementary material available at 10.1007/s40279-025-02243-0.

## Key Points

**Table:** 

Community rugby stakeholders have diverse perspectives regarding concussion risk and the need for prevention, which may impact decisions around participation as well as attitudes towards prevention initiatives. While the emphasis on good management is promising, strategies to reduce risk should also be prioritised.
Multi-pronged initiatives that consider the range of beliefs present in the rugby community are needed to address these views explicitly. This includes carefully translating research evidence in a way that can facilitate buy-in from the community.

## Introduction

Concussions and the potential long-term health consequences associated with this injury have become a concern for all major contact team sports [1, 2]. Rugby union (henceforth rugby) is one of the contact team sports with the highest incidence of concussions [3]. In New Zealand, rugby is played by approximately 137,527 males and females (NZR National Rugby Database, 2022) and accounts for 53% of all sports-related concussions in adults (over 16 years of age) [4]. In school and community senior level players, concussion incidence rates of 3–4 concussions per 1000 player-match-hours and 6–8 concussions per 1000 player-match-hours have been reported for males and females, respectively [5]. However, the true incidence may be greater owing to a high level of non-disclosure [6–8]. In New Zealand high school players, rates as high as 24.7 per 1000 player-match-hours (for females) and 15.5 per 1000 player-match-hours (for males) have been reported (NZR National Rugby Database, 2022). These rates are slightly lower than those recently reported as part of Canada’s Surveillance in High School and Community Sport to REDuce Concussions (SHRed) studies (37.5 per 1000 match-hours in females and 22.0 per 1000 match-hours in males) [9, 10].

In recent years, cases of retired professional athletes and the potential for long-term neurocognitive consequences associated with repetitive head impacts sustained during sporting careers have become a prominent topic in the media and public [11]. Similarly, a high degree of misrepresentation regarding concussion-related research can also be found in the media [12]. Nonetheless, greater awareness of the potential consequences of concussions has led to increased efforts by sporting organizations towards the prevention and management of concussions [2, 13]. Although awareness has improved, the actual translation of concussion guidelines into real-world application in the community setting remains challenging [14]. To address this challenge, New Zealand Rugby (NZ Rugby) developed and implemented a community concussion initiative to support the prevention and management of concussions [15]. This included the creation of a dedicated concussion-management pathway (CMP) with the aim to better support concussion management in community clubs and schools [16]. The goal of the CMP, specifically, is to enhance concussion recognition and facilitate diagnosis, recovery and safe return to play following medical clearance. This pathway’s development was informed by a systems-thinking approach, which considered the role of various levels of stakeholders, from policy to medical support to interpersonal interactions between coaches, parents and players [17, 18].

Since the implementation of the CMP, an iterative process of research, modification and development has taken place [2]. Gaining a deeper understanding of underlying perceptions that inform attitudes and drive behaviours related to concussions and their management is critical to support the future success of such programs [19, 20]. Qualitative research can play a vital role in programme evaluation by providing in-depth insights into the perspectives, experiences and needs of individuals affected by policies and interventions [21]. Understanding how stakeholders within the community rugby system view concussion-related risk and the need for prevention may help us tailor interventions and programmes that not only address the needs of the community but can also work to address problematic beliefs around concussions [22]. Therefore, this study aimed to explore community rugby stakeholder perceptions of concussion risk and the need for prevention. These findings will be used to inform the CMP and its concussion management strategies to ensure the program considers ‘real-world’ challenges [22].

## Methods

### Design

This study reports on one component of a broader programme of work exploring the implementation of the CMP [23–25]. In this study, we adopted a descriptive, qualitative approach to understand experiences through the perspectives and words of the people involved [26]. Our study is situated within a pragmatic paradigm, which aligns with our goal of providing practical findings that may inform policy and practice [27]. From this perspective, multiple realities exist, with these realities being created as individuals interact with their social world [27]. Similarly, we as researchers bring our values, beliefs and attitudes that inevitably influence the way we view the world and how we interpret and approach our research. As a research team, we are all passionate about rugby and dedicated to the welfare of players. Approval to conduct the research was obtained from the University of Otago Human Ethics Committee (approval number 18/087). All participants provided informed written consent. Written consent was also obtained from their parents/caregivers for players aged 15 years or younger.

### Participants and Data Collection

Purposive sampling was used to recruit stakeholders who were actively participating in the CMP from male and female schools (players aged 13–18 years) and club teams (≥ 19 years). Stakeholders included provincial union^1^ representatives, players, coaches, school/club sport administrators, team leads (managers), physiotherapists, nurses and doctors from four provincial unions in NZ. Sampling took place during the 2022 community season. A total of *n* = 62 participants were included. Semi-structured interviews (*n* = 22) and focus groups (*n* = 12) were used to understand stakeholders’ experiences of the CMP (Table 1) and to explore perceptions around concussion risk, current prevention strategies and the need for prevention. The combination of both methods of data collection reflected a pragmatic approach and was employed to facilitate maximum inclusion of participants across different stakeholder groups.

**Table 1:** Sample description (*n* = 62)

Stakeholders	*N*	Age, years		Sex		Level	
		Mean	Range	Male	Female	School	Club/premier
Team leads/managers	4	50.3	31–63	2	2	3	1
Coaches	8	42.3	33–61	7	1	4	4
Parents	3	49.3	41–53	2	1	3	0
Physiotherapists	6	27.6	23–38	4	2	1	5
Players	23	17.3	13–25	14	9	13	10
Provincial Union representatives	6	41	32–54	5	1	–	–
School contacts	5	48.8	24–68	3	2	5	0
GPs	6	50.6	32–66	4	2	–	–
Nurses	1	38	–	0	1	1	0
Total	62			41	21		

Interviews and focus groups were held at a familiar location (i.e. school or rugby club) at a convenient time for the participants and lasted between 40 and 75 min. All interviews and focus groups were audio-recorded for analyses at a later stage. Additional information regarding the collection of data is contained in Supplementary Material 1.

### Analysis

NVivo 14 (Lumivero, CO, USA) was used to organise data. Reflexive thematic analysis was used to analyse the data [28, 29]. This process was undertaken by the first author, with input from the wider team. As a first step, we read each transcript to become familiar with the data and to compile notes about potential codes. Thereafter, data were coded inductively. A subset of transcripts was also coded by a second coder with the aim to generate discussion around codes and their meaning. Codes across all stakeholder groups were sorted and collated into themes and subthemes and presented for the sample as a whole. As part of the results, individual stakeholder groups were highlighted if specifically relevant as part of the theme. An iterative team discussion process was utilised to explore codes and potential themes within the data. Themes were considered in relation to the coded data extracts and the overall dataset. At this stage, a thematic map was developed, and the generated themes were reviewed in regular research team meetings until the team was satisfied that the themes contained a comprehensive description of the data. As is accepted convention within qualitative research, we use terms such as ‘the majority of participants’; ‘many participants’; ‘a few number of participants’ to signify the distribution of the theme within the sample [30]. However, in line with Braun and Clarke [30], we believe the ‘keyness’ of a theme is not necessarily dependent on quantifiable measures (i.e. the number of participants) but rather on whether it captures something important in relation to the overall research question.

We adopted a relativist perspective towards rigor and trustworthiness, recognizing that the criteria for evaluation are study specific and influenced by context [31]. In this respect, it was important to us to design and conduct a study that could contribute in a practical way to concussion prevention and management for NZ Rugby. Similarly, it was important to us that the findings were explored in an iterative manner. Thus, regular team meetings were held to discuss the analysis process and findings, to test assumptions and to facilitate coherence in our interpretation.

Regarding saturation, we concur with Braun and Clarke [32] that data saturation is not a universally useful concept for all types of qualitative research. Our approach to this study aligns with the views of Braun and Clarke (2006; 2019) and Low (2019) that new insights may be developed as long as data are being collected [30, 32, 33]. As such, our focus was on a reflexive and iterative analysis of the data collected [31].

## Results

**Fig. 1:**
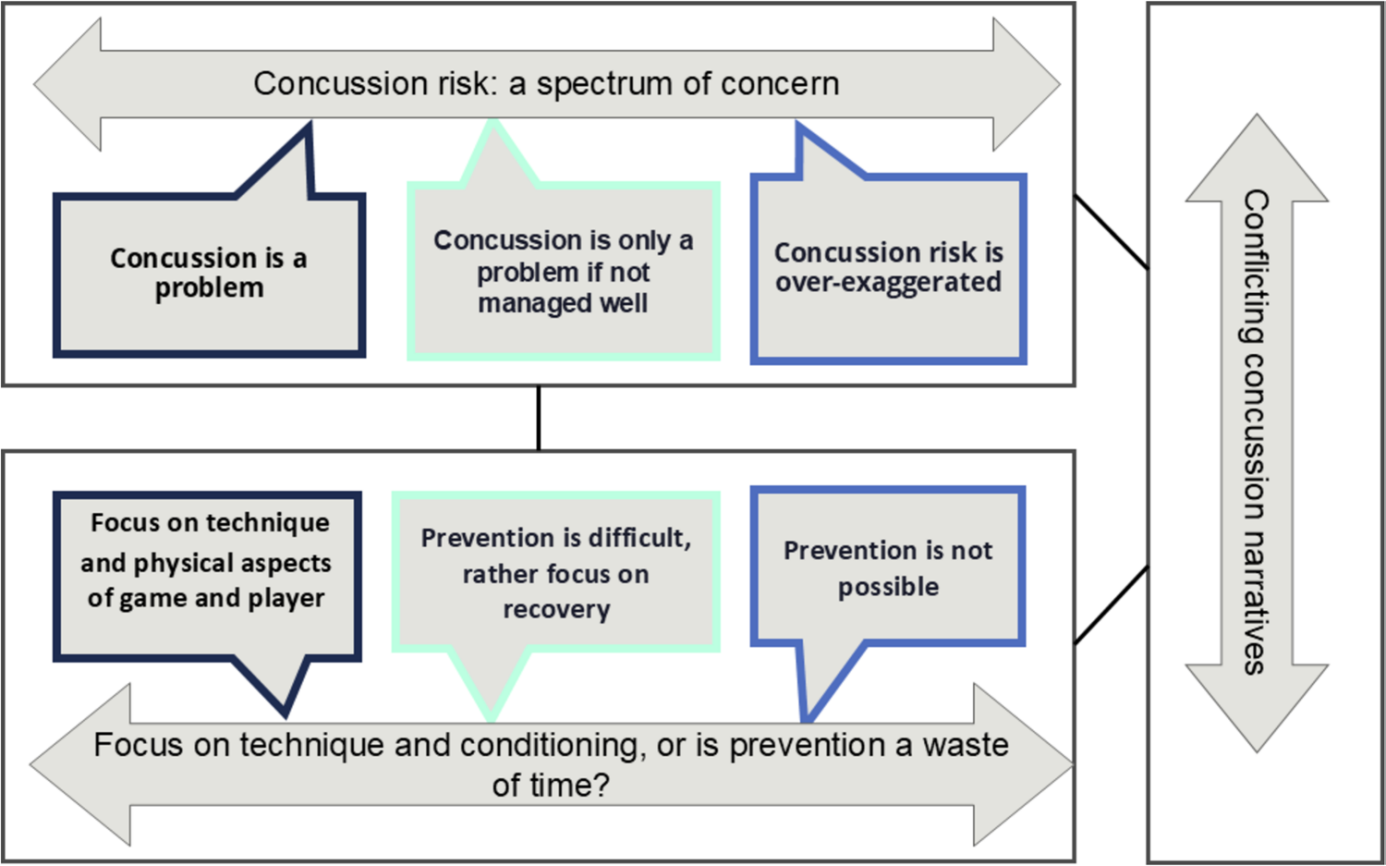
Participant perceptions of concussion-related risk and prevention

A total of three major themes were identified in the data (Fig. 1). Participants had diverging views around the extent of the concussion problem, the risks associated with concussions and their perception for the need for prevention.

### Concussion Risk: a Spectrum of Concern

The majority of participants across stakeholder groups believed concussion awareness, management and disclosure have improved in recent years and that people are taking concussions more seriously:

Concussion management across the board has improved significantly over the past five or so years. Awareness of it is getting a whole lot better and people are actually taking it seriously nowadays (A6 physiotherapist interview).

However, although awareness improved, perceptions around the consequences of heightened concussion awareness and the ‘true risk’ varied. These varying perceptions were grouped according to three subthemes.

#### Concussions Are a Problem

Several participants believed that, as an injury, concussions were the greatest concern for the sport and its participants because of their high prevalence and the difficulty associated with recognising and managing theses injuries. Participants also felt that the problem was exacerbated by some people not taking concussions seriously enough and that the rugby community still lacked adequate understanding of concussions.

I know heaps, probably five or six Premier and Colt’s players combined, that were unconscious and then went back to play next week, because they had no one to stop them, which was unfortunate to see. And it’s obviously a problem because of the effects that we’re seeing later on down the track (C6 physiotherapist focus group).

You’re starting at zero knowledge with these guys. They see it all in Super Rugby and whatever that concussions happen, and players are out, but they don’t understand what that actually means and... and especially the school kids, they think they’re all All Blacks already, so they have to get back to rugby, it’s so important. I’m trying and to let them understand that actually what’s going on here is far more important than rugby (B5 GP focus group).

Overall, this perspective often appeared to be strongly influenced by participants’ fears and concerns around the potential of serious long-term consequences associated with concussions and the awareness created around this in the media.

It is scary. There was that England player, he’s only 40 or something, and he won the World Cup and he said, “I would hand back all of those rugby memories just to feel normal again”. That hit home a little bit. And there’s [a] NZ player who’s no good as well with early onset dementia (A13 player interview).

But it’s not really realized until later in life. You don’t see it instantly, which is perhaps why in the past we didn’t do much about it, because you could see a broken leg, but you couldn’t really see a broken head. It wasn’t until Carl Hayman for example, what is he 40 something? With dementia. We haven’t seen the results of those head injuries till a number of years later (D4 parent interview).

Participants across stakeholder groups also noted that due to the seriousness of concussion, ‘we must do everything we can to keep our players safe’ (A8 school contact) and that risking long-term consequences was not worth it, as there is ‘more to life than rugby’ (A5 physiotherapist interview). Questions were also raised around ‘how many hits are too many’ (C8 parent interview).

#### Concussions are a Problem Only if not Managed Well

This subtheme centred around the perspective identified in several participants that concussions are a problem only if they are not managed well. As part of this perspective, participants believed concussions are going to happen but that the risk associated with these injuries is due to poor management. These participants reiterated that compared with what happened in the past, we now know a lot more about concussions, and we understand the importance of managing them correctly. Thus, the focus has shifted to reducing the impact of injury and ensuring recovery before returning to play:

I remember my parents telling me about an All Black who came forward with early onset dementia, he scored a game-winning try in the World Cup and can’t recall it. He’s pretty young for dementia to come on. That’s pretty hard to hear. But in those times, if you got knocked out in a game, you would take 5 minutes on the bench, go back on. So it doesn’t worry me now because the things are in place, and our understanding is better, it doesn’t scare me to not play, because we have way more understanding and knowledge of concussions now (C4 player focus group).

In addition, most felt that it was important to be informed about the health risks associated with concussions but that people should also take responsibility for risk by ensuring they report their concussions and recover properly before returning to play. Some participants reiterated that all injuries need to be respected and be managed correctly.

I think there’s got to be a realisation that if you play contact sport, there are injuries involved in those sports, and concussion is one of them. I think what is important, is looking after it, having robust protocols, and I think a lot more information to player’s parents, as well (A11 team lead).

Less concern was placed on long-term consequences, as participants believed that those currently coming forward with neurodegenerative conditions come from a rugby playing era where concussions were disregarded owing to a lack of knowledge and management was not always ideal or was non-existing. In this sense, participants believed the risk posed currently when concussions are managed well is not the same as the historic risk experienced. Participants also noted examples of current elite players who are taking concussion management seriously and ensuring they are looking after themselves.

Look at how [name of current All Black player] just self-reported his own concussion. He was out three weeks. Didn’t worry about the expectations a country had on him. He just did what’s best for him (D1 player interview).

However, a few participants acknowledged that they felt that if a player was to sustain multiple concussions, this could pose a risk for long-term health.

#### Concussion Risk is Over-Exaggerated

As part of this theme, participants believed that concussions have always been part of the game and that we are just more aware of them now, which potentially makes it seem like ‘a bigger problem than it really is’ (C3 player interview). Several participants also felt the risk associated with concussions in the elite game is not the same as at the community game. However, what happens in the elite game, nonetheless, negatively drives community perceptions.

One thing I don’t like, and I don’t know if it’s social media or the TV coverage, is the fact that when we see a head knock in the professional game it’s replayed ten times. I think rugby league plays it once and don’t show it again, and I think that would be beneficial, we don’t need to keep seeing it. It happened we need to identify it, but we don’t want to scare people away from the game, what we are watching is a professional game, it’s not the game that the eight-year old are playing in the park. But the parents see that and go, I don’t want my son and daughter to be playing because that’s what’s going to happen to them (C9 Provincial Union (PU) representative interview).

Participants were of the opinion that the true problem with concussions was the fact that it is now ‘over-emphasised’, people are hyper-vigilant and players are getting ‘over-managed’ for every potential head knock. This phenomenon was especially evident for coaches of younger age groups, who feared repercussions should they miss an injury that turns out to be a concussion owing to the increasing scrutiny on the issue. Overall, they felt these perceptions were detrimental to the game and that these would negatively impact participation rates. They felt the over-awareness and ‘spotlight on concussion’ (C9 PU representative interview), especially in rugby, posed a danger to losing the essence of the game.

Concussions are probably deterring a lot of people, particularly kids, from playing the game. We’ve been able to get over shoulder, leg, knee injuries for a long time, but this concussion thing is, definitely from a perception point of view, probably changing the game. Particularly with schools, there’s a lot of publicity around head knocks and concussions that puts people off the game. I think our participation numbers are dramatically affected by the impacts of concussion and the light that’s shone on the topic at the moment (D6 coaches focus group).

These participants felt that the awareness and media attention around concussions have led to people to make some causal links between sustaining a concussion and the development of neurocognitive disease later in life, while the positive physical and mental aspects of sport participation are being overlooked.

There’s a heightened worry around the safety of the game. But if anything, there’s more being done to make it safer than ever… as opposed to the massive long-term effects on our health and our system of a kid doing nothing, as opposed to those kids that are physically active and getting injured. That’s a hard thing to quantify because you can see injuries, where you can’t see the inactivity and the long-term effect of that (A12 PU representative interview).

If you get a concussion you’re going to get dementia, it feels like this is the narrative that is getting pushed at the moment, and it’s not true… it's all about, “Any contact’s bad contact”. I think there needs to be a bit more awareness that concussions just don’t come from rugby. And the guys that are getting the early onset dementia are mostly professional guys, and they’ve gone through that era where it wasn’t really recognized (D6 coach focus group).

Players who were aligned with this perspective often mentioned that although they thought what they were seeing in the media around long-term consequences was ‘scary’ (A1 player focus group), they did not think the same could happen to them, as they were not exposed to the same level of risk. For some players, it was difficult to think about or worry about ‘things in the future’ (A4 player focus group), and they expressed that playing now and living in the moment was much more important.

That is pretty scary. It’s something that’s always in the back of your mind. But if you’ve got a passion for the sport, you just got to try your best to avoid it really. There's nothing really you can do about it other than making the game softer, but you don’t really want to do that (B1 player interview).

### Focus on Technique and Conditioning, or is Injury Just the Nature of the Game?

Participants’ perceptions of and their approach towards prevention efforts were grouped in three diverse subthemes.

#### Focus on Technique and Physical Aspects of the Game and the Player

Several participants believed concussions were a result of poor tackling technique and coaching. Others also noted that a lack of physical conditioning, strength and fitness, as well as contact readiness, were responsible for concussions, especially at the start of the season. Participants also noted that they thought players were getting ‘bigger and faster’ and that weight and age discrepancies in opposing players contributed to concussion incidence. Several participants aligned with this perspective by placing importance on primary prevention efforts (i.e. prevention efforts aimed at preventing concussions from happening in the first place). This included efforts to improve technique and safety, especially with regard to tackling.

We do a lot around the contact area. That’s probably the one thing that I pride myself on as a coach, you can always talk concussions, but trying your best to prevent them is the best thing (C11 coach interview).

I think we don’t do enough preseason training. We go into contact being unprepared, having not doing enough contact work at trainings and before warmup. My guess is that this is how most of our injuries happen, especially the first half of the season. Pre-season is a big thing you have to prepare for contact (B2 player interview).

However, in all coach interviews, the lack of time to properly incorporate technique training was unanimously raised.

Certainly, at the level that we’re all coaching in regard to the club or school, is that you’ve got a limited amount of time with these players, that’s all you've got. Sometimes it may only be once a week during the week, and it could only be an hour and a half, if you’re lucky you’ll get two of those in a week plus the weekend. So, you’re limited in what you can do. So, I suppose that you’re looking at the best thing overall for your team rather than looking at individual technique (C12 coach interview).

Participants also felt that incorporating strength and conditioning, and proper warm-up routines, could also help prevent concussions. A few participants mentioned protective gear as a valuable tool for prevention, while some emphasised the importance of enforcing the rules of game, referee education and law changes to make the game safer. Several participants mentioned the need for improved technical knowledge and skills for coaches, more coach development opportunities and efforts to ensure technique is taught correctly from a young age and that different aspects of technique are taught at the right time points in players’ development. In addition, concussion education that would upskill players around how poor tackle technique could lead to concussions and which situations could pose a danger was recommended. One coach felt strongly that, as a sport, rugby needs to start thinking about how we can regulate contact, especially during training.

#### Prevention is Difficult, Rather Focus on Recovery

In contrast to the first prevention theme, other participants placed their focus on secondary prevention efforts (i.e. preventing secondary injuries and other health consequences once the concussion has occurred by focusing on optimal management and good recovery). For many of these participants, complete mitigation of concussions was difficult, if not impossible, and instead, they placed more importance on early, efficient management (recognise, remove, recover and return) of concussions once they had been sustained.

Well, a lot of the concussions are kind of unavoidable even from certain contacts. So I guess, if it’s unavoidable, the focus has to be in the recovery (C4 player focus group).

It’s a reality, if we’re going to continue to play this game, there will be head clashes and there’s going to be knocks to the head because it’s a confrontational game, we can’t really prevent that. So, I don’t know if that’s a problem, it’s just something, how best can we best manage it? And I think what’s happened for us here in the last three years, we’ve made huge strides and gains, and I think we are looking after our players better than we’ve ever had in the past (C5 school contact interview).

I think at the moment like most of the healthcare system, we’re an ambulance at the bottom of the cliff, rather than trying to prevent anything. So, I think the big thing at the moment is just trying to get those guys to recognize that it is an issue, and it could be an issue long-term, so actually take it seriously. And then obviously taking people through the return-to-play side of things. I don’t know how you switch that from being a, “You’ve got a concussion, let’s help you get back” (C6 physiotherapist interview).

This included the importance of continued education for all stakeholders on the importance of managing concussions well, ensuring players were fully recovered and that they had been medically cleared before returning to play:

I don’t think there’s anything you can do as a coach for an accident or friendly fire where it’s actually your own player’s head that you’ve run into or own player’s knee. You can’t educate against that. Players need to understand the reasons why you have to have that 21-day stand-down and go through the return-to-play protocol. (C8 parent interview).

#### Prevention is not Possible

Some participants reiterated the fact that rugby is a contact sport, and ‘contact’ was seen to inevitably lead to concussions, regardless of tackle technique or physical conditioning. Some participants therefore believed that it is not possible to prevent concussions, unless the game was changed significantly to remove impacts (an option which many participants were not in favour of).

There’s always going to be risks involved with a high impact sport like rugby. Rules like lowered tackle height I think just take away from the game. You just got to mitigate it as much as you can, but you can’t start ruining the game for it because then people just won’t be interested in playing (A13 player interview).

Within this theme, participants often reported not having a specific approach to concussion prevention and that teaching tackle technique was taught for many different reasons, not for just preventing concussion per se.

### Conflicting Concussion Narratives

Importantly, several participants adopted aspects of more than one perspective, and, on the basis of the context being discussed, their perspectives would shift. From a risk-acceptance perspective, some believed concussions were unavoidable; however, they nonetheless valued the importance of risk avoidance behaviours such as prevention efforts directed towards tackle technique.

In the heat of the moment, it just happens, but when you can prevent it by warming up properly, and the proper drills and stuff during training this is important (A4 player focus group).

Alternatively, for example, several players were concerned about the consequences but accepted the risk nonetheless or accepted and managed the risk on the basis of certain conditions:

I like playing rugby so, you sort of accept the risk. Injuries do happen, like concussions will happen. It doesn’t change my perspective on playing rugby. I know the risks and it’s the same as you’ll see in boxing and other sports where they get the head knocks. There is risk involved… If I was to get multiple concussions and ongoing symptoms, that’s when I would think about it and take a step back but at this stage, it doesn’t affect me at all (C2 player focus group).

Similarly, some reported that concussions are very serious and can have major impacts on one’s health; however, they also feel that there is a sense of over-awareness and hypervigilance around concussion which is detrimental to the game.

For me, our responsibility is to make best attempts to look after the players’ health, wellbeing, welfare, but this comes with more responsibility. Some of the rules that we have around, whilst they’re great, they can also be harmful for a kid as well, because we’re pulling kids off the field, because they knock their ear, or their nose, or they’re just tired and they say they are concussed, because they don’t probably realize that the word concussion is such a taboo word now, at school rugby. And at junior rugby that they are okay to use it, but don’t realize they’re using it incorrectly, it’s 3 weeks out of the game for them (A9 team lead focus group).

The shift in perspectives also pointed towards an internal conflict facing participants around what is acceptable and in what circumstances.

Two years ago, I got concussed, I knew straight away, I got up straight away and acted like nothing happened and played on for another five minutes before limping off with a pretend groin injury. But I didn’t want my season to be over if I felt great the next week, or the week after, because of the mandatory stand-downs that we have in place (C10 coach interview).

In addition, worry was expressed about sustainability of the game when trying to balance both the entertainment value of the game and keeping players safe.

Watch any game, when is the crowd’s most engaged, it’s when there’s a fight or a big hit. That’s what humans are like. How do you balance that? Can we keep kids safe, but also people entertained at the professional end. At what stage do you go from a safety-first community game where the effort and focus is on keeping people safe, into entertainment where people want to see is big hits?... I can see a time where rugby isn’t allowed, in another 10 years if things carry on the same path, you wouldn’t be allowed, as a school, to put kids in a situation where some of them are going to get these symptoms or these injuries (C12 coach interview).

## Discussion

This study aimed to explore community rugby stakeholder perceptions of concussion risk and the need for prevention. Three themes were identified; (1) ‘concussion risk: a spectrum of concern’; (2) ‘focus on technique and conditioning or is injury just the nature of the game?’ and (3) conflicting concussion narratives. Although participants perceived that awareness around concussions has improved, several felt that the awareness and identification of concussions have become over-exaggerated and were having a detrimental impact on the game. Participants described being concerned about concussions and the potential long-term consequences and emphasised the need for prevention. Others believed that prevention is difficult and that rather the focus should be directed towards proper management. It was clear that risk and prevention perspectives were in many respects related; participants who regarded concussions as a major problem were also more risk averse and placed more importance on prevention efforts. In a contrasting perspective, participants attempted to ‘tone down the narrative’, by reporting that, for example, the risk is not the same in the amateur game, concussion will happen regardless of technique and prevention is not necessary.

Varied community perceptions and narratives around sports-related injury risk and consequences is similarly reported in literature. Fuller and Ward explored rugby players and spectators’ perceptions on the acceptability of different rates and severity of injury and reported that, even within the groups, there existed wide variations in the level of risk that was believed to be acceptable [34]. More recent studies in former professional football and rugby players found that ex-players self-report downplaying or disregarding the seriousness of concussions during their careers but now have significant concerns regarding the potential detrimental effects of these injuries [35–37]. Similarly, concerns especially around the acceptability of concussion risk with regards to children has been widely published and debated [38, 39]. In contrast, other research has reported the flip-side of the coin, where parents indicated that they understood the risks related to concussion in their children’s sport and tended to minimalise the risk by comparing it with other sports or activities or by elevating the physical, cognitive and social advantages of participating in sport [40, 41]. For players, non-disclosure due to not wanting to miss out, letting the team down or wanting to win at all cost remains a persistent issue [6, 8, 42, 43]. Several of our findings align with Parry et al. (2022), who describe that people invested in rugby, such as coaches and players, can be resistant to the increased reporting and representation of concussion in the media as a threat to the sport and its existence [44]. In addition, some participants in our study reiterated that injury is just part of the game. In this sense, ‘being tough’ still resonated with many rugby players and community rugby stakeholders, leading them to accept pain and injury as inevitable and part of the sport [44, 45]. Merrick et al. (2023) investigated community perceptions of rugby-related spinal cord injuries and similarly found variability in perceptions, from being overly concerned and cautious to disregarding the risk and worrying instead that the game will be changed through safety-driven changes [46].

Determining what is an acceptable level of injury risk in sport is a challenging issue. Langley and Cryer (2012) suggest that some injuries are accepted as an inevitable part of sports participation; however, an injury that presents a threat to life or of disability is usually considered unacceptable [47]. In this study, participants appeared to judge risk as ‘unacceptable’, ‘acceptable’ and ‘acceptable with a caveat’ (with the necessary measures in place). Determining an ‘acceptable’ level of risk could also involve a comparison with the risk associated with other common sports and non-sporting activities [48]. Fuller (2008) classified the risk of catastrophic injuries in rugby according to the risk standards of the UK Health and Safety Executive, in which risk was categorised as ‘unacceptable’, ‘tolerable’, ‘acceptable’ and ‘neglible’ [48]. However, because concussions are often unreported and therefore undiagnosed, it is difficult to be certain of the magnitude of this risk [49]. Regardless of classifications, evaluations of the ‘acceptability’ of risks are also dependent on the value people place on these risks and are strongly influenced by the pre-existing beliefs of the individual [38, 48].

A range of sources may contribute to an individual’s perception of risk, including personal observations, scientific data, expert opinion, media reports and the views of peers [38]. Tversky and Kahneman’s (1973) ‘availability heuristic’ or ‘availability bias’ implies people rely on immediate examples that come to mind when evaluating a specific subject. Thus, if people can think of an incident in which a certain risk has come to fruition, their perception of its likelihood to occur becomes exaggerated [50]. In this sense, former professional players recently disclosing long-term health consequences appears to have had an important impact on community players’ perception of concussions. It appeared that, in some ways, this elevated the seriousness or concerns some participants had regarding concussions. However, these accounts of former players also had the opposite effect, resulting in others minimizing or downplaying the risk by pointing out the differences between the professional and community game or blaming poor concussion management of these injuries in the past.

The media (including sports commentators) plays an important role in forming public perceptions around concussion, regardless of scientific accuracy [11, 12, 51, 52]. This includes both sensationalising the long-term implications of injury and celebrating the ‘big hits’ and warriors of sports [44]. Rugby governing bodies and unions should consider taking a more proactive stance in the media around what the current evidence states and what steps are being taken to reduce concussion-related risks. In addition, governing bodies should highlight that rugby at a community level is different to the elite game in terms of concussion risk and the management in place to support their return to play. Governing bodies are also uniquely placed to engage in open conversations with the media around their responsibility in providing the viewing public with accurate information surrounding concussion; their description of these incidents may shape public perceptions [52].

The community’s perception of risk has important implications for both the sport overall and for player welfare. Community perceptions of injury risk can impact participation rates [53, 54], a concern that was strongly voiced within this study. Moreover, low levels of perceived risk have been reported as significant psychological risk factors for actual injury [55]. Community perceptions of risk may also influence attitudes and behaviours around prevention efforts [56–58]. It cannot be contested that rugby involves physical contact, which can lead to injuries. From an epidemiological view, concussion incidence rates are high in relation to other rugby injuries and prevention efforts have been a priority for the governing body of the sport [59]. The prevention of concussions and their potential long-term effects were the two top-rated research priorities identified for future concussion research in the most recent consensus statement for concussion in sport [2]. In rugby, the majority of concussions occur in the tackle, making it an important focus for prevention efforts [59–61]. Law changes are one such strategy that have previously shown success in other aspects of the game [62]. Initiatives that focus on addressing the technical aspects of the tackle via coach and player education could also be beneficial in reducing concussion risk if structured, translated and implemented appropriately [59, 60].

In addition, neuromuscular training warm-up programmes have been associated with reductions in concussion rates [63]. Nonetheless, we have to consider that, overall, the evidence is sparse with regard to strategies that may effectively prevent concussion [63]. Primary prevention can significantly impact overall injury incidence and burden of sports injuries; however, no preventive measure will likely ever eradicate injuries [64]. This necessitates a focus on the implementation of evidence-based strategies that contribute to the reduction of injury risk [63, 64]. Fuller’s (2007) risk management approach adopts a concept of risk mitigation that explores the potential for reductions in both the incidence and the severity of injuries [53]. Prevention efforts to reduce injuries are needed; however, similarly reducing the severity or risk of subsequent injury is also an important avenue to mitigate risk related to concussions [64]. In this study, not all participants placed importance on primary prevention efforts. However, it was encouraging that several participants did take the management of concussion seriously and saw this as a way (sometimes the only way) to mitigate risk.

NZ Rugby prioritises reducing the incidence of concussions, as well as safe management of the injury according to the four Rs (recognise, remove, recover, return). Safety initiatives include training coaches to teach safer techniques and educating people on concussion recognition and management. However, several of the themes identified in this study do not align with these goals. As seen in the findings, unfavourable beliefs include downplaying the seriousness of concussion, perceptions that the risk is over-exaggerated, the belief that prevention is not possible or not necessary or the perspective that concussion is only a problem if not managed well. Going forward, NZ Rugby should prioritise and invest in multi-pronged strategies aimed at addressing these perspectives. Good management is critical; however, efforts to reduce the risk are also equally important, and this goal requires dedicated strategies to facilitate buy-in and actual implementation of efforts aimed at reducing concussions.

Injury prevention efforts are only able to reduce the risk of injuries if they are actively adopted by athletes, coaches or other relevant stakeholders [22, 65]. While several participants valued the prevention efforts, others did not believe that concussions could be prevented, and similarly, others believed that focusing on the management of concussions once they occurred was more important. The community’s adoption of an injury prevention initiative is, in part, dependent on the degree to which the initiative is perceived to be valuable and superior to the idea or practice it replaces [66]. Therefore, the community perceptions around prevention identified in this study should be considered in the development of future prevention strategies. Coaches are particularly important, as time invested by coaches around injury prevention influences players’ beliefs and attitudes towards injury prevention [67]. Understanding how end-users think about concussion-related prevention may be critical for the design and implementation of prevention initiatives, whether it be law changes, tackle technique training or strength and conditioning programmes. However, the coaches in this study reported a distinct lack of time to invest into efforts such as tackle training. In conjunction with coach knowledge, this remains a pervasive issue in community rugby [68]. Including coaches as key members in the development of injury prevention strategies is needed to facilitate buy-in [69]. In particular, coaches need to see enough value in prevention efforts to overcome challenges such as ‘not having enough time’. In this sense, a greater emphasis on the ‘performance benefit’ of interventions is needed [70]. An example would be to reframe tackle technique training as a priority, as it will result in the most successful tackles, not just the safest [71].

In addition, evidence suggests that public health interventions that are based on social and behavioural science theories are more effective than those that are not [72]. Previous research utilising the health belief model [72] has shown that perceived social influence and norms and individual-self efficacy, followed by benefits, health cues and barriers, were the behavioural determinants most strongly associated with the intention to participate in injury prevention programmes [73]. The perceived benefit of the action plays an important role, for example, players had stronger intentions to report a concussion if they perceived doing so would help them ‘be better off in the long run’ or ‘return to sport sooner’ [74]. Elite player role models can play a key part in destigmatizing concussion disclosure and promoting risk-reducing behaviours, a strategy that governing bodies should prioritise [75, 76].

As our understanding and research into concussion increases, it is evident that previous generations of rugby players may not have received appropriate or adequate levels of concussion support [36, 37]. Current evidence supports the implementation of concussion management protocols (removal from play, requirements to receive clearance to return to play from a licensed health care professional, and education of coaches, parents, and athletes) [2, 63, 77]. In this sense, NZ Rugby’s CMP plays an important role in keeping players safe to give them a clear pathway and support to manage their concussion. Nonetheless, there is a need to keep the rugby community informed with the best evidence available to assist in changing unfavourable beliefs, with evidence for tackle technique training and the need for optimal management and prevention [61]. Similarly, a careful approach to balancing fears with facts is required, keeping player safety a priority without resorting to fear mongering. Coordinated information across sports is needed: providing the importance of good management, stating the facts and the benefits of managing concussion well, while also impressing the serious consequences if not managed well [61, 78]. The media (including social media, influencers and pro-athletes) have potential to positively influence public perceptions of concussions in this regard, to mediate misconceptions and to increase knowledge on a large scale [51, 79].

Beyond just providing information, this should include carefully translating research evidence in a way that can truly facilitate buy-in from the community. For example, research has shown that reporting skill (knowing the actions to take in reporting) was more important than having knowledge of concussions or concussion symptoms [80]. In this respect, the CMP provides a clear pathway for concussion management, with an identified team lead who players report their concussion to. Finally, if we want to keep growing a safe game, we need to realise these differing perspectives may influence interventions and how these interventions are received. When we develop injury prevention and management interventions, we need to take into account that the rugby community ‘comes to the table’ with widely differing beliefs.

This study has certain limitations. The total number of participants within certain individual stakeholder groups were relatively small. Although the aim of the study was to highlight themes within a community rugby system, further in-depth exploration of individual stakeholder groups will be useful to expand on these findings and identify potential differences between groups. Furthermore, the number of participants in the respective focus groups were relatively small, which may limit variety in views. However, the overall number of focus groups were substantial (*n* = 12), and with the addition of individual interviews, we believe we have captured a broad range of views according to what was practically and pragmatically possible during data collection. Finally, we do not assert our findings as the only possible understandings of stakeholders’ perceptions of concussion-related risk and prevention. Instead, our aim was to produce a coherent description of participants’ experiences relevant to our research question. As this study only included participants that were part of the CMP, these findings do not necessarily encompass the concussion perceptions of those who are outside of the structures of the CMP, or in other sports. In this sense, future research is needed to expand on these findings.

## Conclusions

The findings from this study highlight a spectrum of concussion-related perspectives from a range of rugby stakeholders that has important implications for the field of injury prevention and management. Several participants believed that concussions as an injury were the greatest concern for the sport. In line with this perspective, several placed emphasis on the importance of prevention efforts. However, contrasting perspectives were also evident. Several argued that rugby, being a contact sport, inevitably involves concussions and that the current concerns about their risks are exaggerated. These conflicting beliefs may act as a barrier to the successful implementation of interventions to reduce risk. Multi-pronged initiatives that consider the range of beliefs present in the rugby community are needed to address these views explicitly.

## Supplementary Information

Below is the link to the electronic supplementary material.

Supplementary file1 (DOCX 25 KB)

Supplementary file2 (DOCX 39 KB)

## Footnotes

1 Regional rugby union governing body.

## Declarations

### Availability of Data and Materials

The datasets analysed during the current study are available from the corresponding author on reasonable request.

## References

[1] Malcolm D. The impact of the concussion crisis on safeguarding in sport. Front Sports Act Living. 2021;3:42. 10.3389/fspor.2021.589341. 10.3389/fspor.2021.589341 PMC7947280 33718866

[2] Patricios JS, Schneider KJ, Dvorak J, et al. Consensus statement on concussion in sport: the 6th International Conference on Concussion in Sport-Amsterdam, October 2022. Br J Sports Med. 2023;57:695–711. 10.1136/bjsports-2023-106898. 37316210 10.1136/bjsports-2023-106898

[3] Van Pelt KL, Puetz T, Swallow J, et al. Data-driven risk classification of concussion rates: a systematic review and meta-analysis. Sports Med. 2021;51:1227–44. 10.1007/s40279-021-01428-7. 33721284 10.1007/s40279-021-01428-7

[4] Theadom A, Starkey NJ, Dowell T, et al. Sports-related brain injury in the general population: an epidemiological study. J Sci Med Sport. 2014;17:591–6. 10.1016/j.jsams.2014.02.001. 24602688 10.1016/j.jsams.2014.02.001

[5] worldrugby.org. Injury Surveillance Research |World Rugby. https://www.world.rugby/the-game/player-welfare/research/injury-surveillance (accessed 30 Jan 2024)

[6] Salmon D, Mcgowan J, Sullivan SJ, et al. What they know and who they are telling: concussion knowledge and disclosure behaviour in New Zealand adolescent rugby union players. J Sports Sci. 2020;38:1585–94. 10.1080/02640414.2020.1749409. 32264762 10.1080/02640414.2020.1749409

[7] Fraas MR, Coughlan GF, Hart EC, et al. Concussion history and reporting rates in elite Irish rugby union players. Phys Ther Sport Off J Assoc Chart Physiother Sports Med. 2014;15:136–42. 10.1016/j.ptsp.2013.08.002. 10.1016/j.ptsp.2013.08.002 24035483

[8] Roberts SP, Trewartha G, England M, et al. Concussions and head injuries in English community rugby union match play. Am J Sports Med. 2017;45:480–7. 10.1177/0363546516668296. 28146395 10.1177/0363546516668296

[9] West SW, Shill IJ, Sick S, et al. It takes two to tango: high rates of injury and concussion in ball carriers and tacklers in high school boys’ rugby. Clin J Sport Med. 2023;33:405. 10.1097/JSM.0000000000001118. 36633403 10.1097/JSM.0000000000001118

[10] Shill IJ, West SW, Sick S, et al. Injuries and concussions in female high school rugby: prevention is worth a try. Clin J Sport Med. 2022;32:508–16. 10.1097/JSM.0000000000000993. 34759178 10.1097/JSM.0000000000000993

[11] Jeckell AS, Fontana RS, Gonzalez R. Review of media representation of sport concussion and implications for youth sports. Clin Sports Med. 2024;43:159–72. 10.1016/j.csm.2023.06.012. 37949509 10.1016/j.csm.2023.06.012

[12] Choi AR, Feller ER. Misrepresentation of mild traumatic brain injury research in press releases. PM&R. 2022;14:769–78. 10.1002/pmrj.12656. 34156765 10.1002/pmrj.12656

[13] Harmon KG, Clugston JR, Dec K, et al. American Medical Society for Sports Medicine position statement on concussion in sport. Br J Sports Med. 2019;53:213–25. 10.1136/bjsports-2018-100338. 30705232 10.1136/bjsports-2018-100338

[14] Fraas MR, Burchiel J. A systematic review of education programmes to prevent concussion in rugby union. Eur J Sport Sci. 2016;16:1212–8. 10.1080/17461391.2016.1170207. 27063067 10.1080/17461391.2016.1170207

[15] Salmon D, Sullivan J, Romanchuk J, et al. Infographic. New Zealand rugby’s community concussion initiative: keeping kiwi communities RugbySmart. Br J Sports Med. 2020;54:300–1. 10.1136/bjsports-2019-100949. 31302602 10.1136/bjsports-2019-100949

[16] Salmon D, Romanchuk J, Murphy I, et al. Infographic. New Zealand Rugby’s concussion management pathway. Br J Sports Med. 2020;54:298–9. 10.1136/bjsports-2019-100950. 31300392 10.1136/bjsports-2019-100950

[17] Clacy A, Goode N, Sharman R, et al. A knock to the system: a new sociotechnical systems approach to sport-related concussion. J Sports Sci. 2017;35:2232–9. 10.1080/02640414.2016.1265140. 27935422 10.1080/02640414.2016.1265140

[18] Hulme A, Thompson J, Plant KL, et al. Applying systems ergonomics methods in sport: a systematic review. Appl Ergon. 2019;80:214–25. 10.1016/j.apergo.2018.03.019. 29674008 10.1016/j.apergo.2018.03.019

[19] Register-Mihalik J, Linnan LA, Marshall SW, et al. Using theory to understand high school aged athletes’ intentions to report sport-related concussion: implications for concussion education initiatives. Brain Inj. 2013;27:878–86. 10.3109/02699052.2013.775508. 23789865 10.3109/02699052.2013.775508

[20] de Vries H. An integrated approach for understanding health behavior; the I-change model as an example. Psychol Behav Sci Int J. 2017;2: 555585. 10.19080/PBSIJ.2017.02.555585.

[21] Bekker S, Bolling C, Ahmed OH, et al. Athlete health protection: why qualitative research matters. J Sci Med Sport. 2020;23:898–901. 10.1016/j.jsams.2020.06.020. 32665215 10.1016/j.jsams.2020.06.020

[22] Alex D, Finch CF. Planning for implementation and translation: seek first to understand the end-users’ perspectives. Br J Sports Med. 2012;46:306–7. 10.1136/bjsports-2011-090461. 22006931 10.1136/bjsports-2011-090461

[23] Salmon DM, Badenhorst M, Keung S, et al. Utilisation of New Zealand Rugby’s concussion management pathway: a mixed methods investigation. Eur J Sport Sci. 2024. 10.1002/ejsc.12213. (**Published Online First: 5 November**). 39500620 10.1002/ejsc.12213 PMC11621389

[24] Salmon D, Badenhorst M, Clark B, et al. Unintended consequences—a qualitative exploration of baseline testing in community rugby concussion management. J Sci Med Sport. 2024. 10.1016/j.jsams.2024.05.003. 38811276 10.1016/j.jsams.2024.05.003

[25] Salmon DM, Walters S, Brown J, et al. Managing concussion in the real world: stakeholder perspectives of New Zealand Rugby’s concussion management pathway. Int J Sports Sci Coach. 2024;19:1515–30. 10.1177/17479541231218518.

[26] Sandelowski M. Focus on research methods: Whatever happened to qualitative description? Res Nurs Health. 2000;23:334–40. 10.1002/1098-240x(200008)23:4%3c334::aid-nur9%3e3.0.co;2-g. 10940958 10.1002/1098-240x(200008)23:4<334::aid-nur9>3.0.co;2-g

[27] Savin-Baden M, Major CM. Qualitative research: an essential guide to theory and practice. London: Routledge; 2013.

[28] Braun V, Clarke V. Can I use TA? Should I use TA? Should I not use TA? Comparing reflexive thematic analysis and other pattern-based qualitative analytic approaches. Couns Psychother Res. 2021;21:37–47. 10.1002/capr.12360.

[29] Braun V, Clarke V. Reflecting on reflexive thematic analysis. Qual Res Sport Exerc Health. 2019;11:589–97. 10.1080/2159676X.2019.1628806.

[30] Braun V, Clake V. Using thematic analysis in psychology. Qual Res Psychol. 2006;3:77–101. 10.1191/1478088706qp063oa.

[31] Burke S. Rethinking ‘validity’ and ‘trustworthiness’ in qualitative inquiry. How might we judge the quality of qualitative research in sport and exercise sciences? In: Smith B, Sparkes A, editors. Routledge handbook of qualitative research in sport and exercise. Routledge; 2016.

[32] Braun V, Clarke V. To saturate or not to saturate? Questioning data saturation as a useful concept for thematic analysis and sample-size rationales. Qual Res Sport Exerc Health. 2021;13:201–16. 10.1080/2159676X.2019.1704846.

[33] Low J. A pragmatic definition of the concept of theoretical saturation. Sociol Focus. 2019;52:131–9. 10.1080/00380237.2018.1544514.

[34] Fuller CW, Ward CJ. An empirical approach for defining acceptable levels of risk: a case study in team sports. Inj Prev J Int Soc Child Adolesc Inj Prev. 2008;14:256–61. 10.1136/ip.2008.018739. 10.1136/ip.2008.018739 18676785

[35] Walton SR, Kerr ZY, Mannix R, et al. Subjective concerns regarding the effects of sport-related concussion on long-term brain health among former NFL players: an NFL-LONG study. Sports Med Auckl NZ. 2022;52:1189–203. 10.1007/s40279-021-01589-5. 10.1007/s40279-021-01589-5 34773581

[36] Daly E, White A, Blackett AD, et al. Pressure. A qualitative analysis of the perception of concussion and injury risk in retired professional rugby players. J Funct Morphol Kinesiol. 2021;6:78. 10.3390/jfmk6030078. 34564197 10.3390/jfmk6030078 PMC8482162

[37] Daly E, Blackett AD, Pearce AJ, et al. Protect the player, protect the game: reflections from ex-professional rugby union players on law changes, protective equipment, and duty of care in the professional game. J Funct Morphol Kinesiol. 2022;7:91. 10.3390/jfmk7040091. 36278752 10.3390/jfmk7040091 PMC9624300

[38] Quarrie KL, Brooks JHM, Burger N, et al. Facts and values: on the acceptability of risks in children’s sport using the example of rugby-a narrative review. Br J Sports Med. 2017;51:1134–9. 10.1136/bjsports-2017-098013. 28724697 10.1136/bjsports-2017-098013

[39] Piggin J, Batten J, Parry K, et al. Compulsory collisions and corporate interests in school rugby: challenging distortions in the framing of childhood injury. Inj Prev. 2022. 10.1136/ip-2022-044775. 36376056 10.1136/ip-2022-044775

[40] Anderson E, White A, Hardwicke J. A qualitative exploration of parents’ perceptions of risk in youth contact rugby. Behav Sci. 2022;12:510. 10.3390/bs12120510. 36546993 10.3390/bs12120510 PMC9774146

[41] McGlynn J, Boneau RD, Richardson BK. “It might also be good for your brain”: cognitive and social benefits that motivate parents to permit youth tackle football. J Sport Soc Issues. 2020;44:261–82. 10.1177/0193723520903226.

[42] Salmon D, Badenhorst M, Walters S, et al. The rugby tug-of-war: exploring concussion-related behavioural intentions and behaviours in youth community rugby union in New Zealand. Int J Sports Sci Coach. 2022;17:804–16. 10.1177/17479541211047661.

[43] Foster CA, D’Lauro C, Johnson BR. Pilots and athletes: different concerns, similar concussion non-disclosure. PLoS One. 2019;14: e0215030. 10.1371/journal.pone.0215030. 31042725 10.1371/journal.pone.0215030 PMC6493720

[44] Parry K, White AJ, Cleland J, et al. Masculinities, media and the rugby mind: an analysis of stakeholder views on the relationship between rugby union, the media, masculine-influenced views on injury, and concussion. Commun Sport. 2022;10:564–86. 10.1177/21674795211027292.

[45] Liston K, McDowell M, Malcolm D, et al. On being ‘head strong’: the pain zone and concussion in non-elite rugby union. Int Rev Sociol Sport. 2018;53:668–84. 10.1177/1012690216679966.

[46] Merrick N, Badenhorst M, Morgan A, et al. Community perspectives on spinal cord injury in rugby union: facts and fears. Sci Med Footb. 2023. 10.1080/24733938.2023.2253191. 37650220 10.1080/24733938.2023.2253191

[47] Langley J, Cryer C. A consideration of severity is sufficient to focus our prevention efforts. Inj Prev. 2012;18:73–4. 10.1136/injuryprev-2011-040278. 22294564 10.1136/injuryprev-2011-040278

[48] Fuller CW. Catastrophic injury in rugby union: is the level of risk acceptable? Sports Med. 2008;38:975–86. 10.2165/00007256-200838120-00002. 19026015 10.2165/00007256-200838120-00002

[49] Reider B. Full disclosure. Am J Sports Med. 2018;46:19–21. 10.1177/0363546517744756. 29283282 10.1177/0363546517744756

[50] Tversky A, Kahneman D. Availability: a heuristic for judging frequency and probability. Cognit Psychol. 1973;5:207–32. 10.1016/0010-0285(73)90033-9.

[51] Ahmed OH, Hall EE. “It was only a mild concussion”: exploring the description of sports concussion in online news articles. Phys Ther Sport Off J Assoc Chart Physiother Sports Med. 2017;23:7–13. 10.1016/j.ptsp.2016.07.003. 10.1016/j.ptsp.2016.07.003 27639135

[52] Ku C, McKinlay A, Grace RC, et al. An international exploration of the effect of media portrayals of postconcussion management on concussion identification in the general public. J Head Trauma Rehabil. 2020;35:218–25. 10.1097/HTR.0000000000000547. 31834064 10.1097/HTR.0000000000000547

[53] Fuller C. Managing the risk of injury in sport. Clin J Sport Med. 2007;17:182–7. 10.1097/JSM.0b013e31805930b0. 17513908 10.1097/JSM.0b013e31805930b0

[54] Creyer E, Ross W, Evers D. Risky recreation: an exploration of factors influencing the likelihood of participation and the effects of experience. Leis Stud. 2003;22:239–53. 10.1080/026143603200068000.

[55] Kontos AP. Perceived risk, risk taking, estimation of ability and injury among adolescent sport participants. J Pediatr Psychol. 2004;29:447–55. 10.1093/jpepsy/jsh048. 15277587 10.1093/jpepsy/jsh048

[56] Verhagen E, van Stralen M, van Mechelen W. Behaviour, the key factor for sports injury prevention. Sports Med. 2010;40:899–906. 10.2165/11536890-000000000-00000. 20942507 10.2165/11536890-000000000-00000

[57] Rimal RN, Real K. Perceived Risk and Efficacy Beliefs as motivators of change: use of the risk perception attitude (RPA) framework to understand health behaviors. Hum Commun Res. 2003;29:370–99. 10.1111/j.1468-2958.2003.tb00844.x.

[58] Yoo S-W, Kim J, Lee Y. The effect of health beliefs, media perceptions, and communicative behaviors on health behavioral intention: an integrated health campaign model on social media. Health Commun. 2018;33:32–40. 10.1080/10410236.2016.1242033. 27858470 10.1080/10410236.2016.1242033

[59] Hendricks S, Emery C, Jones B, et al. ‘Tackling’ rugby safety through a collective approach. Br J Sports Med. 2023;57:562–3. 10.1136/bjsports-2023-107020. 37045555 10.1136/bjsports-2023-107020

[60] Cross MJ, Tucker R, Raftery M, et al. Tackling concussion in professional rugby union: a case–control study of tackle-based risk factors and recommendations for primary prevention. Br J Sports Med. 2019;53:1021–5. 10.1136/bjsports-2017-097912. 29021244 10.1136/bjsports-2017-097912

[61] Batten J, White AJ, Anderson E, et al. From management to prevention: the new cure for sports concussion. Br J Sports Med. 2016;50:1293–4. 10.1136/bjsports-2015-095949. 27215936 10.1136/bjsports-2015-095949

[62] Hendricks S, Lambert MI, Brown JC, et al. An evidence-driven approach to scrum law modifications in amateur rugby played in South Africa. Br J Sports Med. 2014;48:1115–9. 10.1136/bjsports-2013-092877. 24550209 10.1136/bjsports-2013-092877

[63] Eliason PH, Galarneau J-M, Kolstad AT, et al. Prevention strategies and modifiable risk factors for sport-related concussions and head impacts: a systematic review and meta-analysis. Br J Sports Med. 2023;57:749–61. 10.1136/bjsports-2022-106656. 37316182 10.1136/bjsports-2022-106656

[64] Verhagen E, Clarsen B, van der Graaff L, et al. Do not neglect injury severity and burden when assessing the effect of sports injury prevention interventions: time to paint the whole picture. Br J Sports Med. 2024;58:1166–9. 10.1136/bjsports-2024-108215. 38969484 10.1136/bjsports-2024-108215 PMC11503035

[65] CarolineF F. A new framework for research leading to sports injury prevention. J Sci Med Sport. 2006;9:3–9. 10.1016/j.jsams.2006.02.009. 16616614 10.1016/j.jsams.2006.02.009

[66] Hendricks S, Tucker R, Paul L, et al. Applying diffusion innovation theory to evaluate the attributes of the new tackle law in rugby football codes. Br J Sports Med. 2024;58: bjsports-2024. 10.1136/bjsports-2024-108376. 10.1136/bjsports-2024-108376 38580398

[67] Hendricks S, den Hollander S, Lambert M. Coaching behaviours and learning resources; influence on rugby players’ attitudes towards injury prevention and performance in the tackle. Sci Med Footb. 2020;4:10–4. 10.1080/24733938.2019.1633470.

[68] Dane K, Foley G, Hendricks S, et al. “It’s always the bare minimum”—a qualitative study of players’ experiences of tackle coaching in women’s rugby union. J Sci Med Sport. 2023;26:149–55. 10.1016/j.jsams.2023.01.002. 36669901 10.1016/j.jsams.2023.01.002

[69] Garnett D, Cobbing S, Viljoen C, et al. High school rugby coaches’ knowledge and opinions of concussion in Kwa-Zulu Natal province in South Africa: an ecological cross-sectional study. BMC Sports Sci Med Rehabil. 2024;16:139. 10.1186/s13102-024-00930-5. 38915113 10.1186/s13102-024-00930-5 PMC11194973

[70] Minnig MC, Hawkinson L, Root HJ, et al. Barriers and facilitators to the adoption and implementation of evidence-based injury prevention training programmes: a narrative review. BMJ Open Sport Exerc Med. 2022. 10.1136/bmjsem-2022-001374. 36187085 10.1136/bmjsem-2022-001374 PMC9516217

[71] Burger N, Lambert M, Hendricks S. Lay of the land: narrative synthesis of tackle research in rugby union and rugby sevens. BMJ Open Sport Exerc Med. 2020;6: e000645. 10.1136/bmjsem-2019-000645. 32518671 10.1136/bmjsem-2019-000645 PMC7254146

[72] Glanz K, Bishop DB. The role of behavioral science theory in development and implementation of public health interventions. Annu Rev Public Health. 2010;31:399–418. 10.1146/annurev.publhealth.012809.103604. 20070207 10.1146/annurev.publhealth.012809.103604

[73] Gabriel EH, Hoch MC, Cramer RJ. Health belief model scale and theory of planned behavior scale to assess attitudes and perceptions of injury prevention program participation: an exploratory factor analysis. J Sci Med Sport. 2019;22:544–9. 10.1016/j.jsams.2018.11.004. 30501955 10.1016/j.jsams.2018.11.004

[74] Weber Rawlins ML, Welch Bacon CE, Tomporowski P, et al. Using the health belief model to predict concussion-reporting intentions and behaviour. Brain Inj. 2020;34:16455–65. 10.1080/02699052.2020.1831069. 33044873 10.1080/02699052.2020.1831069

[75] Salmon DM, Badenhorst M, Brown J, et al. Concussion education for New Zealand high school rugby players: a mixed-method analysis of the impact on concussion knowledge, attitudes and reporting behaviours. Int J Sports Sci Coach. 2024;19:99–112. 10.1177/17479541231156159.

[76] Kerr ZY, Register-Mihalik JK, Haarbauer-Krupa J, et al. Using opinion leaders to address intervention gaps in concussion prevention in youth sports: key concepts and foundational theory. Inj Epidemiol. 2018;5:1–11. 10.1186/s40621-018-0158-7. 29984386 10.1186/s40621-018-0158-7 PMC6035905

[77] Arakkal AT, Barón AE, Lamb MM, et al. Evaluating the effectiveness of traumatic brain injury state laws among high school athletes. Inj Epidemiol. 2020;7:12. 10.1186/s40621-020-00241-6. 32279659 10.1186/s40621-020-00241-6 PMC7153238

[78] Calderwood C, Murray AD, Stewart W. Turning people into couch potatoes is not the cure for sports concussion. Br J Sports Med. 2016;50:200–1. 10.1136/bjsports-2015-095393. 26405112 10.1136/bjsports-2015-095393

[79] Alexander F, Tucker R, Jones B, et al. X as a proxy for tackle safety culture? Sentiment analysis of social media posts on red-carded and yellow-carded tackles during the 2019 Rugby World Cup. BMJ Open Sport Exerc Med. 2023;9: e001756. 10.1136/bmjsem-2023-001756. 37901749 10.1136/bmjsem-2023-001756 PMC10603336

[80] Warmath D, Winterstein AP, Myrden S. Parents and coaches as transformational leaders: Motivating high school athletes’ intentions to report concussion symptoms across socioeconomic statuses. Soc Sci Med. 2022;292: 114559. 10.1016/j.socscimed.2021.114559. 34776287 10.1016/j.socscimed.2021.114559

