# Supplementary material for: Concussion Risk and the Need for Prevention: An Exploration into the Complexity of Community Perspectives in Rugby Union
Source: Sports Med. 2025 Jun 19;55(12):3129–42. doi: 10.1007/s40279-025-02243-0 (PMC12628456; doi:10.1007/s40279-025-02243-0)
Supplement: Supplementary file 1 — Supplementary file1 (DOCX 25 KB) [file 40279_2025_2243_MOESM1_ESM.docx]

**Online Supplementary file A**

Sports Medicine

***Concussion risk and the need for prevention:* an exploration into the complexity of community perspectives in rugby union**

Marelise Badenhorst^1,2^ (ORCID: 0000-0001-8443-9173)

Janelle Romanchuk^3^ (ORCID^:^ 0000-0002-8442-9589)

Danielle Salmon^3^ (ORCID: 0000-0003-1254-8308)

James Craig Brown^2,4,5^ (ORCID: 0000-0002-7778-7783)

Sharief Hendricks^2,4^ (ORCID: 0000-0002-3416-6266)

Simon Walters^1^ (ORCID: 0000-0002-6467-4982)

^1^Sports Performance Research Institute New Zealand, School of Sport and Recreation, Auckland University of Technology, D-88, Private Bag 92006, Auckland 1142, New Zealand

^2^Carnegie Applied Rugby Research (CARR) Centre, Carnegie School of Sport, Leeds Beckett University, Leeds, UK

^3^New Zealand Rugby, PO Box 2172, Wellington, 6140, New Zealand

^4^Division of Physiological Sciences and Health through Physical Activity, Lifestyle and Sport Research Centre, Department of Human Biology, Faculty of Health Sciences, University of Cape Town, Cape Town, South Africa

^5^Institute of Sport and Exercise Medicine, Department of Exercise, Sport and Lifestyle Medicine, Faculty of Medicine and Health Sciences, Stellenbosch University, Stellenbosch, South Africa

**Corresponding Author:**

Marelise Badenhorst
Sports Performance Research Institute New Zealand
School of Sport and Recreation, Auckland University of Technology
D-88, Private Bag 92006
Auckland 1142, New Zealand

[marelise.badenhorst@aut.ac.nz](mailto:marelise.badenhorst@aut.ac.nz)

**Additional information regarding the methods**

**Data collection**

The data collection team consisted of three females and three males involved in the implementation of the CMP in different provincial unions. The research team held BSc, MSc and PhD degrees respectively, and were actively involved in research and clinical activities within the rugby health and welfare field. No individual relationships were established with participants prior to the study. To facilitate consistency between the team members, experienced qualitative researchers (PhDs) who were part of the research advisory team for the broader project, conducted training sessions with the team prior to the interviews and focus groups to discuss interview / facilitation techniques such as the use of probing questions; and paraphrasing to confirm understanding.

Interviews were conducted one-on-one, and similarly, one team member conducted the focus groups. All team members were involved in conducting both interviews and focus groups. Participants were given refreshments during interviews / focus groups, but no other incentives were given to take part. Focus groups consisted of 3-4 participants with similar roles / from the same stakeholder groups (for e.g., players, coaches etc). The nurse participant was included within a focus group with physiotherapists, as they worked at the same school. We opted for smaller groups as these were logistically easier to arrange and would in our opinion be more time efficient for participants. Authors have argued that the group dynamics in smaller groups, still facilitates participant interaction, a distinctive feature of focus group.^1^ Smaller focus groups have also the advantage of greater opportunity for participants to describe their experiences in more depth. In this way, a small group of participants in conversation, still extends the data beyond what would routinely be available in a one-on-one interview. The disadvantage of smaller groups is that it limits the total range of experiences. However, although the number of participants in focus groups were small, our total number of focus groups is large (n=12). Therefore, we believe we have captured a broad range of views.

*Interview /focus group schedule*

A generic outline of the semi-structured interview schedule is presented below. These questions were adapted according to stakeholder group.

1. What type of injury do you think is the biggest concern in rugby? Why?
2. Do you think concussions are a concern in rugby? Why? What do you think should be done about it?
3. Why do you think concussions happen?
4. How do you think you would you approach returning to play after a knee injury compared to a concussion, would it be different? How is it different?
5. Often concussions are not being reported – why do you think this happens? Do you think this is a problem?
6. Do you have any specific concerns about concussion? Why? What would you like rugby as a sport to do about this? What could you as a coach/player etc. do about it?
7. Do you believe concussions can be prevented? Why/How? What are you/ your team doing to prevent concussion?

**References**

1. Braun V, Clarke V. *Successful Qualitative Research: A Practical Guide for Beginners*. SAGE Publications; 2013. https://books.google.com/books?id=EV_Q06CUsXsC&pgis=1
