# Supplementary material for: Concussion Risk and the Need for Prevention: An Exploration into the Complexity of Community Perspectives in Rugby Union
Source: Sports Med. 2025 Jun 19;55(12):3129–42. doi: 10.1007/s40279-025-02243-0 (PMC12628456; doi:10.1007/s40279-025-02243-0)
Supplement: Supplementary file 2 — Supplementary file2 (DOCX 39 KB) [file 40279_2025_2243_MOESM2_ESM.docx]

**Online Supplementary file B**

Sports Medicine

***Concussion risk and the need for prevention:* an exploration into the complexity of community perspectives in rugby union**

Marelise Badenhorst^1,2^ (ORCID: 0000-0001-8443-9173)

Janelle Romanchuk^3^ (ORCID^:^ 0000-0002-8442-9589)

Danielle Salmon^3^ (ORCID: 0000-0003-1254-8308)

James Craig Brown^2,4,5^ (ORCID: 0000-0002-7778-7783)

Sharief Hendricks^2,4^ (ORCID: 0000-0002-3416-6266)

Simon Walters^1^ (ORCID: 0000-0002-6467-4982)

^1^Sports Performance Research Institute New Zealand, School of Sport and Recreation, Auckland University of Technology, D-88, Private Bag 92006, Auckland 1142, New Zealand

^2^Carnegie Applied Rugby Research (CARR) Centre, Carnegie School of Sport, Leeds Beckett University, Leeds, UK

^3^New Zealand Rugby, PO Box 2172, Wellington, 6140, New Zealand

^4^Division of Physiological Sciences and Health through Physical Activity, Lifestyle and Sport Research Centre, Department of Human Biology, Faculty of Health Sciences, University of Cape Town, Cape Town, South Africa

^5^Institute of Sport and Exercise Medicine, Department of Exercise, Sport and Lifestyle Medicine, Faculty of Medicine and Health Sciences, Stellenbosch University, Stellenbosch, South Africa

**Corresponding Author:**

Marelise Badenhorst
Sports Performance Research Institute New Zealand
School of Sport and Recreation, Auckland University of Technology
D-88, Private Bag 92006
Auckland 1142, New Zealand

[marelise.badenhorst@aut.ac.nz](mailto:marelise.badenhorst@aut.ac.nz)

**Consolidated criteria for reporting qualitative research (COREQ): A 32-item checklist for interviews and focus groups**

| **Section/Topic** | **Item No** | **Checklist item** | **Reported on page No** |
| --- | --- | --- | --- |
| **Domain 1: Research team and reﬂexivity** | | | |
| Personal Characteristics | | | |
| *Interviewer/facilitator* | 1 | Which author/s conducted the interview or focus group? Interviewer/facilitator | Online Supplementary file A |
| *Credentials* | 2 | What were the researcher’s credentials? E.g. PhD, MD | Online Supplementary file A |
| *Occupation* | 3 | What was their occupation at the time of the study? | Online Supplementary file A |
| *Gender* | 4 | Was the researcher male or female? | Online Supplementary file A |
| *Experience and training* | 5 | What experience or training did the researcher have? Relationship with participants | Online Supplementary file A |
| Relationship with participants | | | |
| *Relationship established* | 6 | Was a relationship established prior to study commencement? | Online Supplementary file A |
| *Participant knowledge of the interviewer* | 7 | What did the participants know about the researcher? e.g. personal goals, reasons for doing the research | Online Supplementary file A |
| *Interviewer characteristics* | 8 | What characteristics were reported about the interviewer/facilitator? e.g. Bias, assumptions, reasons and interests in the research topic | Online Supplementary file A and Manuscript Page 8 |
| **Domain 2: Study design** | | | |
| Theoretical framework | | | |
| *Methodological orientation and*  *Theory* | 9 | What methodological orientation was stated to underpin the study? e.g. grounded theory, discourse analysis, ethnography, phenomenology, content analysis | Page 8 |
| Participant selection | | | |
| *Sampling* | 10 | How were participants selected? e.g. purposive, convenience, consecutive, snowball | Page 9 |
| *Method of approach* | 11 | How were participants approached? e.g. face-to-face, telephone, mail, email | Page 9 |
| *Sample size* | 12 | How many participants were in the study? | Page 9 |
| *Non-participation* | 13 | How many people refused to participate or dropped out? Reasons? | N/A |
| *Setting of data collection* | 14 | Where was the data collected? e.g. home, clinic, workplace | Page 9 |
| *Presence of non-participants* | 15 | Was anyone else present besides the participants and researchers? | Online Supplementary file A |
| *Description of sample* | 16 | What are the important characteristics of the sample? e.g. demographic data, date | Table 1 |
| Data collection | | | |
| *Interview guide* | 17 | Were questions, prompts, guides provided by the authors? Was it pilot tested? | Online Supplementary file A |
| *Repeat interviews* | 18 | Were repeat interviews carried out? If yes, how many? | N/A |
| *Audio/visual recording* | 19 | Did the research use audio or visual recording to collect the data? | Page 9 |
| *Field notes* | 20 | Were ﬁeld notes made during and/or after the interview or focus group? | N/A |
| *Duration* | 21 | What was the duration of the interviews or focus group? | Page 9 |
| *Data saturation* | 22 | Was data saturation discussed? | Page 10 |
| *Transcripts returned* | 23 | Were transcripts returned to participants for comment and/or correction? | N/A |
| **Domain 3: Analysis and ﬁndings** | | | |
| Data analysis | | | |
| *Number of data coders* | 24 | How many data coders coded the data? | Page 10 |
| *Description of the coding tree* | 25 | Did authors provide a description of the coding tree? | Page 10, Figure 1 |
| *Derivation of themes* | 26 | Were themes identiﬁed in advance or derived from the data? | Page 10 |
| *Software* | 27 | What software, if applicable, was used to manage the data? | Page 10 |
| *Participant checking* | 28 | Did participants provide feedback on the ﬁndings? | N/A |
| Reporting | | | |
| *Quotations presented* | 29 | Were participant quotations presented to illustrate the themes / ﬁndings? Was each quotation identiﬁed? e.g. participant number | Page 11-22 |
| *Data and ﬁndings consistent* | 30 | Was there consistency between the data presented and the ﬁndings? | Page 11-22 |
| *Clarity of major themes* | 31 | Were major themes clearly presented in the ﬁndings? | Figure 1 |
| *Clarity of minor themes* | 32 | Is there a description of diverse cases or discussion of minor themes? | Page 14, 16, 19, 20 |
